# Disruption of Persistent Nociceptive Behavior in Rats with Learning Impairment

**DOI:** 10.1371/journal.pone.0074533

**Published:** 2013-09-11

**Authors:** Yuxin Ma, Shuxing Wang, Yinghong Tian, Lucy Chen, Guoying Li, Jianren Mao

**Affiliations:** 1 MGH Center for Translational Pain Research, Department of Anesthesia, Critical Care and Pain Medicine, Massachusetts General Hospital, Harvard Medical School, Boston, Massachusetts, United States of America; 2 Department of Anatomy, School of Basic Medicine, Guangdong Pharmaceutical University, Guangzhou, Guangdong, China; 3 Department of Anatomy, Zhongshan School of Medicine, Sun Yat-sen University, Guangzhou, China; University of Kentucky Medical Center, United States of America

## Abstract

Despite the subjective nature of pain experience with cognitive and affective dimensions, preclinical pain research has largely focused on its sensory dimension. Here, we examined the relationship between learning/memory and nociceptive behavior in rats with combined learning impairment and persistent nociception. Learning impairment was induced by bilateral hippocampal injection of a mixed Aβ solution, whereas persistent nociception produced in these rats by complete Freund’s adjuvant-induced ankle inflammation. Those rats with learning impairment showed a diminished development of thermal hyperalgesia and mechanical allodynia and a shorter time course of nociceptive behavior without alteration of their baseline nociceptive threshold. In rats with pre-established hyperalgesia and allodynia due to ankle inflammation, bilateral intra-hippocampal injection of cycloheximide (a protein synthesis inhibitor) promoted the earlier recovery of nociceptive behavior. Moreover, expression of Aβ, NR1 subunit of the N-methyl-D-aspartate receptor, and protein kinase Cγ was upregulated, whereas the choline acetyl transferase expression was downregulated, in the hippocampus, thalamus, amygdala, and/or spinal cord of rats with combined learning impairment and persistent nociception. The data indicate that learning impairment could disrupt the response to a state of persistent nociception, suggesting an important role for cognitive maladaptation in the mechanisms of chronic pain. These results also suggest that a preclinical model of combined learning impairment and persistent nociception may be useful to explore the brain mechanisms underlying the transition from acute to chronic pain.

## Introduction

Pain is a complex subjective experience with sensory-discriminative, motivational-affective, cognitive-evaluative dimensions [1,2]. To date, most studies of pain mechanisms have focused on the sensory-discriminative dimension of pain, while less is known regarding the relationship between the cognitive function and pain perception and its role in the transition from acute to chronic pain [3–5]. Clinically, an increasing body of evidence has indicated that pain perception may be altered in patients with dementia such as Alzheimer’s disease (AD) [6,7], an irreversible neurodegenerative disease characterized by the deposition of different forms of beta-amyloid (Aβ) in the brain, cognitive impairment, and memory loss [8,9]. In the clinical setting, AD patients have been shown to receive fewer analgesics possibly because they report less pain than those with intact cognitive function but similar pain conditions [10,11]. These findings are consistent with a neuroimaging study showing the role of brain structures in retrieving autobiographical memories of painful events [12].

Since pain assessment in patients with cognitive and learning impairment is complex [3,13], clinical studies often exclude such patients from participation [14]. Indeed, current pain assessment tools are inadequate to capture the impact of cognitive and learning dysfunction on pain perception, often resulting in under-treatment of pain in patients with cognitive and learning impairment [15–18]. In the present study, we sought to examine a relationship between learning impairment and pain using a combined rat model. In the first experiment, we examined whether learning impairment would be associated with diminished nociceptive behaviors. In the second experiment, we investigated whether disrupting the function of learning and memory by intra-hippocampal administration of cycloheximide, a protein synthesis inhibitor shown to disrupt memory formation and consolidation [19], would alter the recovery time of pre-established nociceptive behaviors.

## Materials and Methods

### Experimental animals

Male Sprague-Dawley rats weighing 250-300g (Charles River Lab, Wilmington, MA) were used. The animal room was 12h dark/light cycle with lights on from 7AM to 7PM. All animals had *ad libitum* access to water and a standard rat diet. The experimental protocol was approved by the Massachusetts General Hospital Institutional Animal Care and Use Committee.

### Induced learning impairment

Aβ1-40 and Aβ1-42 powders (Sigma, 600 µg, 1:1) were dissolved in 300µl sterile artificial cerebrospinal fluid (ACSF) with 1% NH_4_OH. The solution was then mixed and centrifuged at 15000×g for 5min to a final concentration of 2µg/µl, which was incubated at 37^°^C for 6 days to form a mature Aβ solution before its final use.

To inject the Aβ solution into the hippocampal CA1 area, a surgical procedure was performed under sodium pentobarbital (50mg/kg i.p.) anesthesia [20]. A rat was placed onto a stereotaxic frame. The scalp was shaved and sterilized with a beta-dine solution and alcohol and a 1.0cm midline sagittal incision was made to expose the skull. For microinjection, holes (OD 0.6mm) were drilled through the skull (3.3mm posterior to Bregma, 1.6mm lateral to the midline) according to the rat’s brain atlas of Paxinos and Watson [21]. The mixed Aβ solution (10µg/5µl) was injected slowly (0.5µl/min) into the bilateral hippocampal CA1 area (2.8mm ventral to the brain surface) using a 5-µl microsyringe (Hamilton). The needle was left in place for 10min after the injection for solution dispersion before being slowly withdrawn. The hole on the skull was closed with bone wax (ETHICON). The skin wound was applied with antibiotic to prevent infection and closed with wound clips. Sham control rats underwent the same procedure except that sterile ACSF was injected with the same volume as the Aβ solution. Naïve rats did not undergo the surgical procedure, nor received any injection. The location of the intra-hippocampal injection was confirmed at the time of tissue harvest and with immunostaining. Rats with an incorrect injection site and the lack of learning impairment were excluded from final experiment data analysis.

### Ankle joint inflammation

Ankle inflammation was induced by injecting 50µl of complete Freund’s adjuvant (CFA, Sigma) into the right tibio-tarsal joint under 2% isoflurane anesthesia [22]. The right hind paw was held and the fossa of the lateral malleolus of the fibula was located. A 22-gauge needle was inserted vertically to enter the articular cavity between the tibio-fibular and tarsus bone until a loss of resistance was felt. For sham control, incomplete Freund’s adjuvant (IFA, Sigma, as vehicle of CFA) was injected with the same volume and approach. Naïve control rats received neither CFA nor IFA. Both ankles of rats from CFA and IFA groups were inspected after the injection. The inflammation was indicated by redness and swelling of the injected ankle as well as nociceptive behavior (see below), as compared with the contralateral non-injected hind paw.

### Behavioral tests

#### Morris water maze task

A standard water maze task was used with minor modifications [23]. A circular pool (1.3m in diameter and 75cm in depth) was partially filled with milked water of 25 ± 1^°^C and 30cm in depth. A rat was able to rest for 30 s once it found and climbed onto a platform (12cm×12cm).

Both hidden-platform test and visible-platform test were carried out. The hidden-platform test was carried out on day 8 after the intra-hippocampal Aβ injection (designated as day 1). The process consisted of two trials per day for 5d. During each trial, rats were released from four assigned starting points and allowed to swim for a maximum of 120s. The time elapsed before a rat climbed onto the platform submerged 1cm below the water surface and located in the middle of the southeast quadrant of the water pool (i.e., escape latency from the maze) was recorded. If a rat failed to find the platform in 120s, it would be led to the platform and also allowed to stay for 30s. To rule out the impact of a non-specific effect on the Morris water maze test, the visible-platform test was performed on day 7 and 8 (i.e., days 14 and 15 after the Aβ injection). For this test, the platform was elevated 1cm above the water surface but placed in a different site away from that used for the hidden-platform test. Similarly, the time required to escape onto the visible platform (i.e., escape latency) was recorded. In all tests, rats were placed into the pool facing the wall at the beginning of each trial.

#### Locomotor activity

A rat was placed on a floor and any gait abnormalities were assessed and recorded using the method of Chatani et al. [24]: 0, normal; 1, slightly limping; 2, clearly limping but useful in walking; 3, severely limping and not useful in walking.

#### Thermal hyperalgesia and mechanical allodynia

Two daily 60-min sessions were used to habituate rats to the test environment. The withdrawn latency (in seconds) to thermal stimulation and withdrawal threshold (in grams) to mechanical stimulation were examined for both ipsilateral and contralateral hind paws before (baseline) and at 1, 3, 5, 7, and 14 days after CFA or IFA injection. The thermal hyperalgesia test was performed using the method of Hargreaves et al. [25]. The radiant heat source was adjusted to result in baseline latencies of around 12s and a cut-off latency of 20s. Two trials were performed on each hind paw with a 5min interval. A set of von Frey filaments was used to measure mechanical allodynia using an up-and-down method [26]. Rats were individually placed in a plastic box with a wire mesh floor. A single von Frey fiber was applied to the plantar surface for five times with an inter-stimulation interval of 5s. A positive response was defined as at least one clear paw withdrawal response out of the five applications [26]. All behavioral tests were carried out between 9 AM and noon.

### Immunohistochemistry

Rats were deeply anesthetized with sodium pentobarbital (60mg/kg i.p.), perfused transcardially with 0.9% saline followed by 4% paraformaldehyde in 0.1M phosphate buffer (pH7.4, 4^°^C), brain and lumbar spinal cord segments were removed, post-fixed overnight 4^°^C, then cryoprotected in 30% sucrose in 0.1M phosphate buffer saline (PBS) at 4^°^C until tissue blocks sank to the bottom. Sections (brain: 40µm; spinal cord: 25µm) were cut using a cryostat and washed with 0.01M PBS for 10min×3, blocked for 1h in PBS containing 1% BSA, 0.3% Triton X-100 at room temperature, and then incubated for overnight at 4^°^C with one of the following primary antibodies: Beta-amyloid (Abcam): 1:500, rabbit polyclonal; NR1 receptor - NR1 (Abcam): 1:500, rabbit polyclonal; protein kinase Cγ- PKCγ (Abcam): 1:500, rabbit polyclonal; choline acetyl transferase - ChAT (Chemicon; Millipore): 1:100, rabbit polyclonal. Sections were incubated for 1h at room temperature with FITC- or Cy3-conjugated secondary antibody (1:300; Chemicon, Temecula, CA) and then washed with PBS for 10min×3. For controls, primary antibody was omitted. Sections were examined with a fluorescence microscope (Olympus, Japan), and images were captured with a digital camera and analyzed with Adobe Photoshop version 7.0.

### Western Blot

Rats were sacrificed by decapitation under sodium pentobarbital anesthesia (60 mg/kg i.p.). Brain (hippocampus, amygdala, and thalamus) and spinal cord samples, which were divided into ipsilateral and contralateral side, were removed and rapidly frozen on dry ice and stored at -80^°^C until use. Samples were homogenized in SDS buffer containing a mixture of protease inhibitors (Sigma). Protein samples were prepared on SDS-PAGE gels (4-15% gradient gel) and transferred to polyvinylidene difluoride membranes (Millipore). Membranes were blocked with 5% non-fat dried milk for 1h at room temperature and incubated overnight at 4^°^C with one of the following primary antibodies: Beta-amyloid (Abcam): 1:500, rabbit polyclonal; NR1 (Abcam): 1:1000, rabbit polyclonal; PKCγ (Abcam): 1:250, rabbit polyclonal; ChAT (Chemicon; Millipore): 1:4000, rabbit polyclonal. Membranes were incubated for 1h at room temperature with HRP-conjugated secondary antibody (1:8000; Amersham Biosciences, Arlington Heights, IL). Blots were visualized in ECL solution (NEN, Boston, MA) for 2min and exposed to hyperfilms (Amersham Biosciences) for 0.5-10min. Blots were again incubated in stripping solution (67.5mM Tris, pH 6.8, 2% SDS, and 0.7% β-mercaptoethanol) for 30 min at 50^°^C and reprobed with anti-β-actin antibody (1:12000, mouse monoclonal; Abcam) as loading control. All western blot analyses were made in triplicates.

### Experimental design

#### Experiment 1

**Figure 1:**
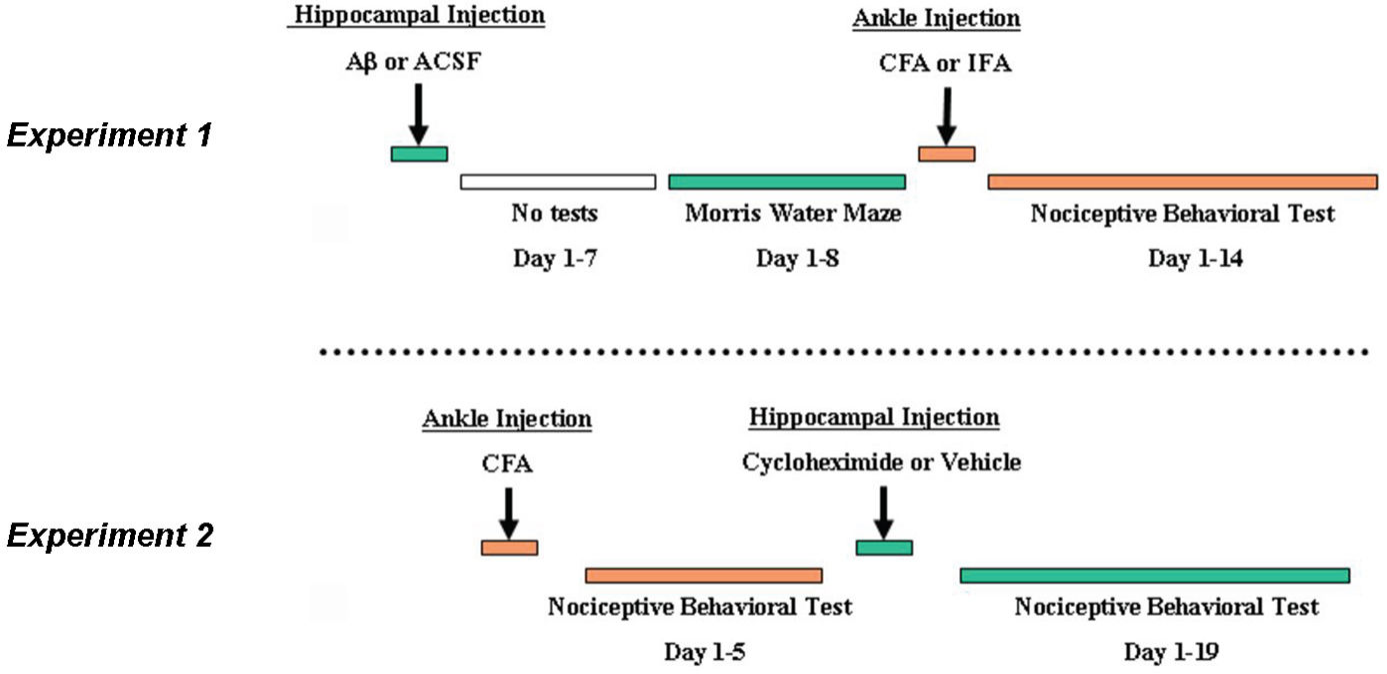
A schematic presentation of experimental designs. **A)** Experiment 1 examined the effect of intra-hippocampal Aβ-induced learning impairment on the development of nociceptive behavior following ankle inflammation. **B**) Experiment 2 examined the effect of intra-hippocampal cycloheximide on the recovery of established nociceptive behavior following ankle inflammation.

(Figure 1A) To examine the effect of learning impairment on the development of nociceptive behavior, the Aβ or ACSF solution was first injected into the bilateral CA1 area of the hippocampus. The Morris water maze test began on day 8 (designated as test day 1) after the hippocampal injection to examine whether learning impairment was induced. The Morris water maze test consisted of two paradigms: 1) the hidden-platform test was carried out over 5 daily sessions from test day 1 to 5, and 2) the visible-platform test was performed in on test day 7 and 8 to rule out non-specific effects. On day 15 after the initial intra-hippocampal injection (i.e., after the last water maze test), either CFA or IFA was injected into the right ankle joint after obtaining the baseline thermal and mechanical nociceptive threshold. The test for nociceptive behavior was then made on day 1, 3, 5, 7, and 14 after the CFA or IFA injection. Accordingly, a total of five experimental groups were included (n=6): Naïve group (no hippocampus or ankle injection), ACSF (hippocampus)/vehicle (ankle) group, ACSF (hippocampus)/CFA (ankle) group, Aβ (hippocampus)/vehicle (ankle) group, and Aβ (hippocampus)/CFA (ankle) group. After the final behavioral test, the Morris water maze test was repeated to confirm the continuing presence of induced learning impairment.

#### Experiment 2

(Figure 1B) To examine the effect of learning impairment on the recovery of nociceptive behavior, cycloheximide (1% DMSO in saline; Sigma-Aldrich) was injected into the hippocampus according to the following experimental protocol. Rats first received the CFA (50µl) injection into the right ankle joint and the nociceptive behavioral test was performed on day 0 (baseline) and post-CFA injection day 1 (designated as day -1), 3 (day -3), and 5 (day -5). On day 0 (i.e., 5 days after the CFA injection), these rats were divided into 2 subgroups (n=6) and received either cycloheximide (30 µg/5µl) or vehicle (5µl) into the bilateral hippocampal CA1 area. After the cycloheximide or vehicle injection, the nociceptive test was repeated on day 1, 3, 5, 9, 13, 17, 19 to compare the time course for the recovery of nociceptive behavior between the cycloheximide and vehicle group.

To confirm the effect of cycloheximide on learning and memory, a group of naïve rats without ankle joint inflammation was used. Rats in this group first underwent the Morris water maze test from day 1 to day 5 (designated as day -1 to -5). On day 0, these rats were divided into 2 subgroups (n=6) and received either cycloheximide (30 µg/5µl) or vehicle (5µl) into the bilateral hippocampal CA1 area. After the cycloheximide or vehicle injection, two more series of the Morris water maze test (5 sessions per series) were performed on day 1 to day 5 and day 15 to day 19 to determine the effect of cycloheximide on the performance of the Morris water maze task. All behavioral tests were carried out during a similar time period on each test day for all groups by an observer who was blinded to the group assignment.

### Statistical analysis

The data from the nociceptive behavioral test was first analyzed using repeated measure two-way ANOVA (group by time) to detect overall significance among groups using SPSS 16.0 for Windows. The *post hoc* Tukey test was performed to detect the source of differences among groups. For Western blot, the density of each band was measured using Photoshop and Quantity One and normalized against the corresponding loading control and the group difference was compared using one-way ANOVA. For the Morris water maze, one-way ANOVA or Student *t*-test was used to detect the group difference. The data are presented as mean± SD (standard deviation).

## Results

### Impaired acquisition of spatial learning by the hippocampal Aβ injection

**Figure 2:**
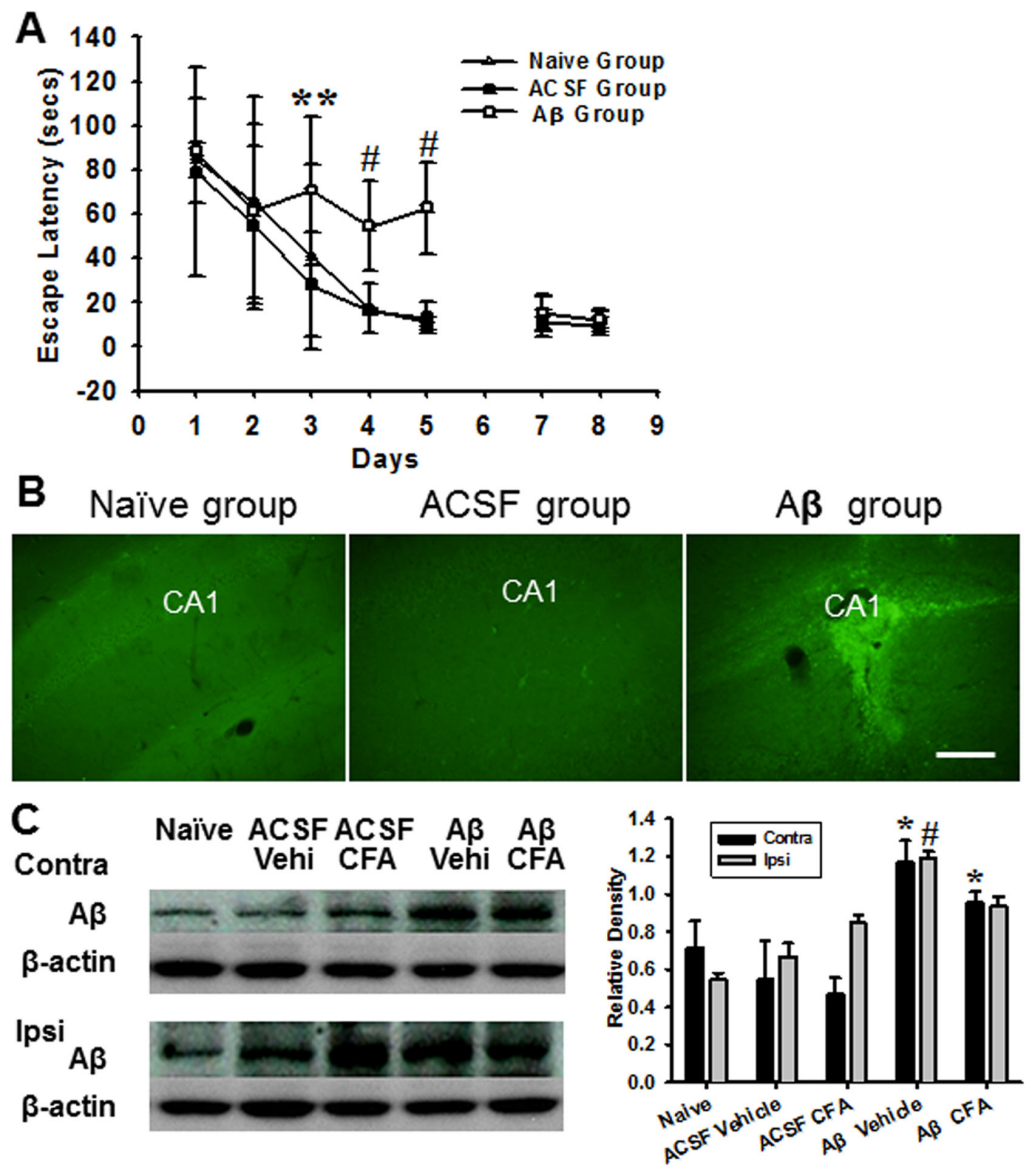
Morris water maze test and hippocampal Aβ expression. **A)** No differences were detected among three groups of rats in the hidden-platform test on day 1 and day 2. However, rats in Aβ group showed a longer escape latency than rats in ACSF group on days 3-5 and rats in naïve group on day 4-5. ** *P*<0.01, as compared with ACSF group; # *P*<0.01, as compared with both naïve and ACSF groups. In contrast, no differences in the escape time in visible-platform test on day 7 and 8. **B)** Immunohistochemical examination of the hippocampal CA1 area showed a substantially increased reactivity of Aβ1-40/1-42 in Aβ group, whereas Aβ immunoreactivity was barely detectable in rats of naïve and ACSF groups at the same hippocampal site. Scale bar: 100 µm. **C)** Western blot revealed a significant increase in Aβ expression in hippocampus contralateral to ankle inflammation in rats receiving Aβ injection with or without ankle inflammation. * *P*<0.05, as compared with contralateral (contra) side of other groups; #*P<*0.05, as compared with ipsilateral (ipsi) side of remaining groups.

The effect of the hippocampal Aβ injection on acquisition of spatial learning was tested by the Morris water maze. While no differences were detected among three groups in the hidden-platform test on day 1 and day 2, rats in the Aβ group showed a significantly longer escape latency than rats in ACSF group on day 3-5 and in ACSF and naïve groups on day 4-5 (Figure 2A, P < 0.01).

Results from two additional tests indicated that the prolonged escape latencies in the hidden-platform test for rats in the Aβ group were due to a learning impairment because 1) rats in the Aβ group had moderately increased latencies to reach a target quadrant in the water pool during a probe test as compared with rats in the ACSF and naive groups (data not shown), and 2) the escape latencies in the visible-platform test on day 7 and day 8 did not differ among rats in all three groups (*P*>0.05). In addition, rats in all three groups exhibited normal gait (scored 0) throughout the experimental period without any indication of locomotor dysfunction or poor swimming performance.

Immunohistochemical examination of the hippocampal CA1 area showed a substantially increased reactivity of Aβ1-40/1-42 in the Aβ group when examined after the last Morris water maze test (Figure 2B). In contrast, Aβ immunoreactivity was barely detectable in rats from the naïve and ACSF groups at the same hippocampal area (Figure 2B). Western blot also showed a significant increase in the Aβ expression on the contralateral (to CFA-injection) hippocampus of rats receiving both Aβ injection and ankle inflammation as compared with naïve rats and rats with ACSF injection with or without inflammation (Figure 2C, P < 0.05). Moreover, the Aβ expression was also significantly increased in the ipsilateral (to CFA-injection) hippocampus in rats receiving both Aβ and CFA injection as compared with naïve rats (Figure 2C, P < 0.05).

Collectively, the combined morphological and behavioral data indicate that the intra-hippocampal Aβ injection induced the upregulated Aβ expression and the impairment of spatial learning in these rats.

### Attenuation of the development of nociceptive behavior in rats with learning impairment

**Figure 3:**
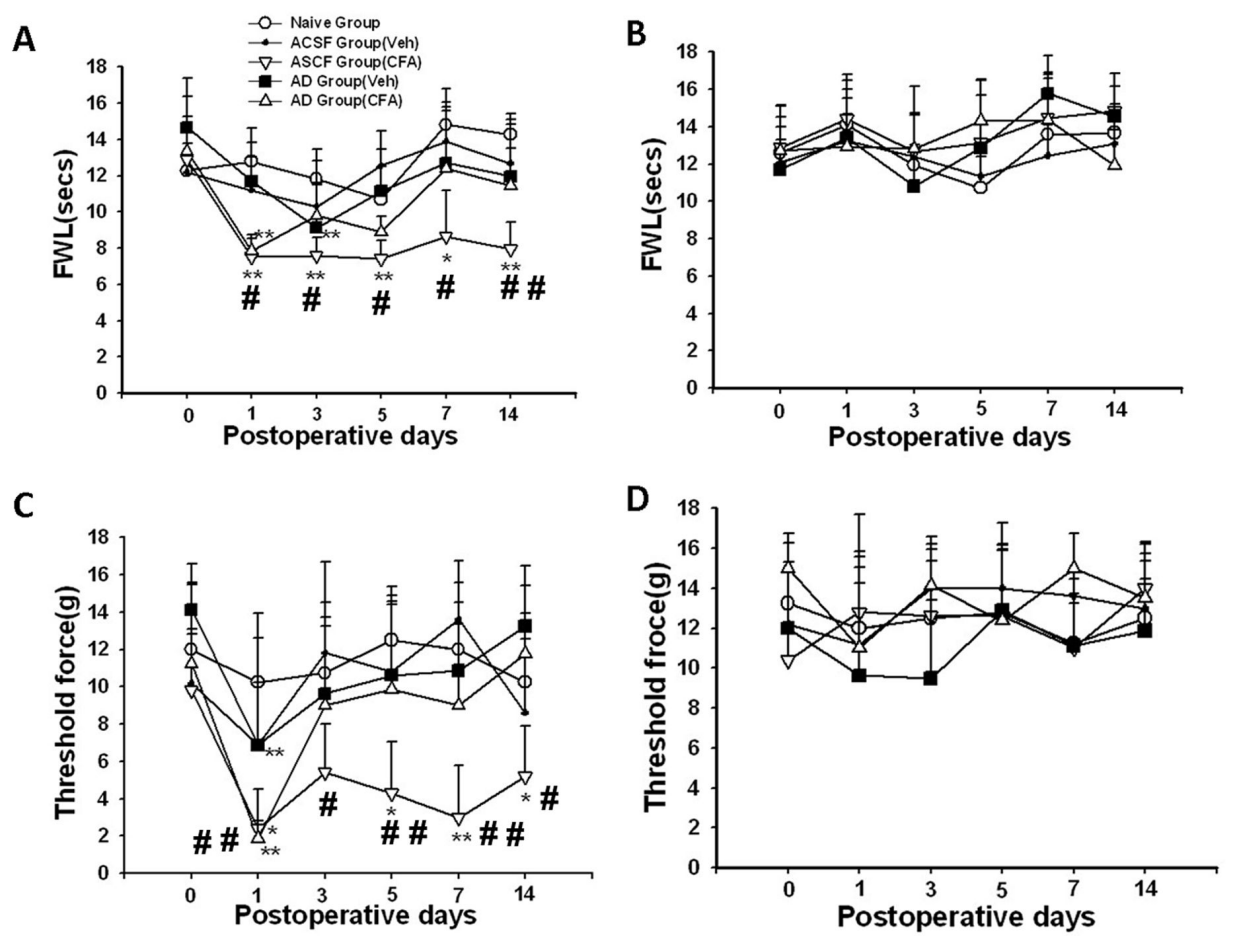
Attenuated nociceptive behavior in rats with learning impairment. **A**, **C**) The development of thermal hyperalgesia (A) and mechanical allodynia (C) was attenuated in Aβ-injected rats. **B**, **D**) No differences were detected in thermal (B) and mechanical (D) nociceptive threshold on contralateral hind paw in all groups. * *P*<0.05, ** *P*<0.01, as compared with baseline threshold of the same group. FWL: foot-withdrawal latency. #<0.05, # # *P*<0.01, as compared with each of the remaining groups at the same time point.

The baseline nociceptive threshold to radiant heat or mechanical (von Frey filament) stimulation did not differ among all groups of rats with or without learning impairment. Thermal hyperalgesia was detected on the ipsilateral hind paw on day 1 after CFA injection in both Aβ and ACSF groups, as compared with rats without ankle inflammation (naïve or vehicle group) (Figure 3A, P < 0.05). In those rats with Aβ-induced learning impairment, thermal hyperalgesia was no longer detectable on day 3 after CFA injection and throughout the remaining experimental period (Figure 3A, P > 0.05). In contrast, thermal hyperalgesia continued to be present over the next two weeks in those rats without learning impairment (Figure 3A, P < 0.05).

Similar results were obtained in the mechanical allodynia test such that rats with learning impairment showed a substantially earlier return to the baseline beginning on day 3 after CFA injection as compared with the rats without learning impairment (Figure 3C, P < 0.05). No significant differences in the thermal and mechanical nociceptive threshold were observed on the contralateral hind paw among all groups of rats during the experimental period (Figure 3B,D, *P*>0.05). Collectively, the data indicate that the development of nociceptive behavior (thermal hyperalgesia and mechanical allodynia) was attenuated in those rats exhibiting learning impairment.

### Expression of ChAT in the hippocampus, thalamus, and amygdala

**Figure 4:**
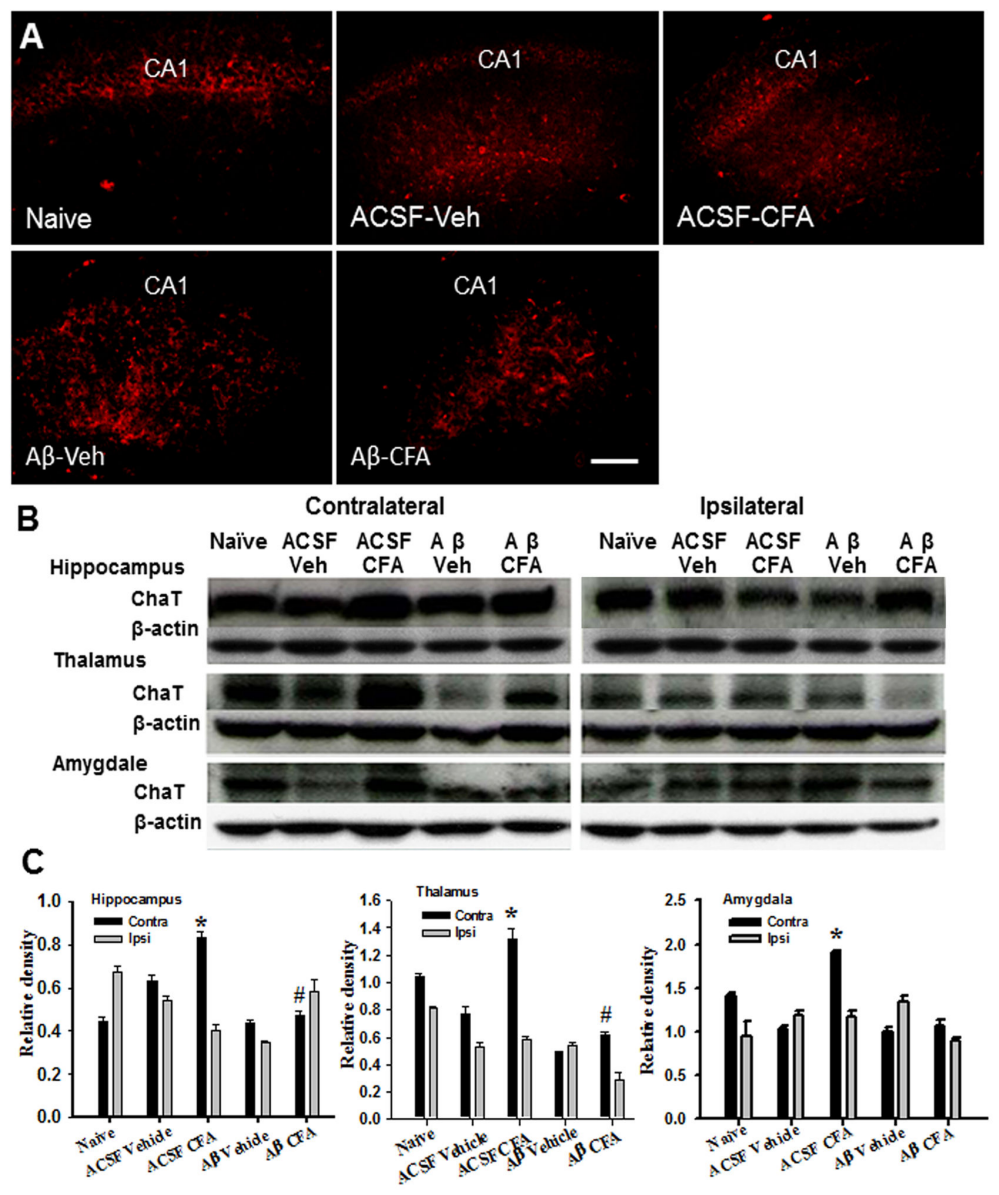
Expression of ChAT in the hippocampus, thalamus, and amygdala. **A**) ChAT immunoreactivity was decreased in the hippocampus contralateral to ankle inflammation in Aβ group as compared with both naïve and ACSF groups with or without ankle inflammation. Veh: Vehicle. Scale bar: 100 µm. **B**, **C**) Western blot showed a substantial downregulation of ChAT expression in the contralateral hippocampus, thalamus, and amygdala of rats in Aβ group with ankle inflammation. * *P*<0.05, as compared with Aβ/vehicle and Aβ/CFA groups; # *P*<0.05, as compared with the ASCF/vehicle group.

Choline acetyl transferase (ChAT) is a cholinergic marker protein for the functional state of cholinergic neurons and its deficit has been associated with conditions of learning impairment seen in AD [27]. Consistently, ChAT immunoreactivity was decreased in the hippocampus contralateral to the inflamed ankle in Aβ group as compared with both naïve and ACSF groups with or without ankle inflammation (Figure 4A). Western blot results also showed a substantial downregulation of ChAT expression on contralateral hippocampus, thalamus, and amygdala of rats in Aβ group with ankle inflammation, as compared with naïve and/or ACSF groups with or without inflammation (Figure 4B,C, each *P*<0.05).

### Expression of NR1 and PKCγ in the spinal cord and brain regions

**Figure 5:**
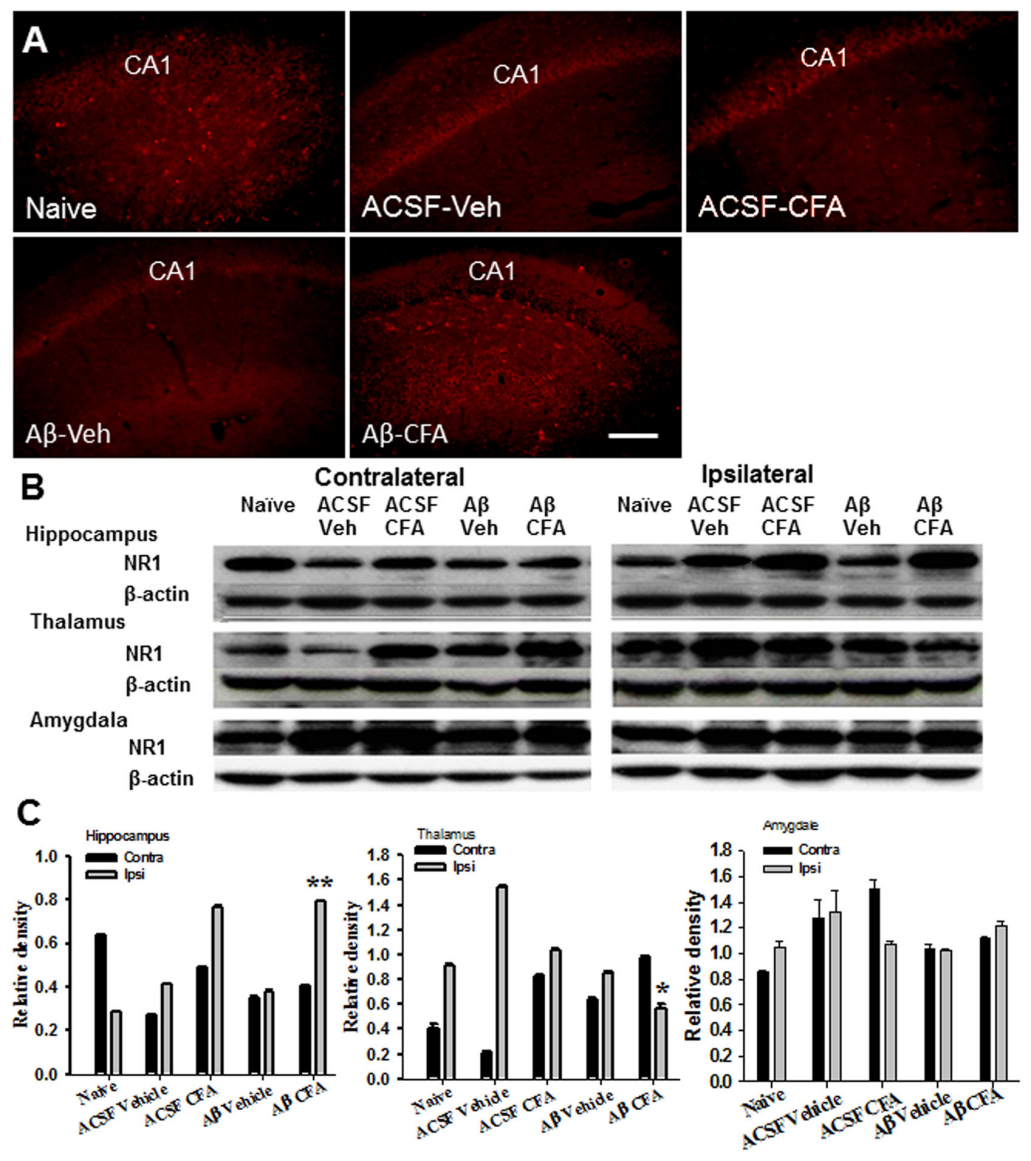
Expression of NR1 in brain regions. **A**–**C**) Immunostaining (A) and Western blot (B, C) showed an upregulation of NR1 on the ipsilateral side (to ankle inflammation) of the hippocampus in rats with the intra-hippocampal Aβ injection. Scale bar: 100 µm. **B**, **C**) In these same Aβ-injected rats, NR1 expression was downregulated in the ipsilateral thalamus but not amygdala. * *P*<0.05 and ** *P*<0.01, as compared with the naïve and ACSF group on the same (ipsilateral) side.

**Figure 6:**
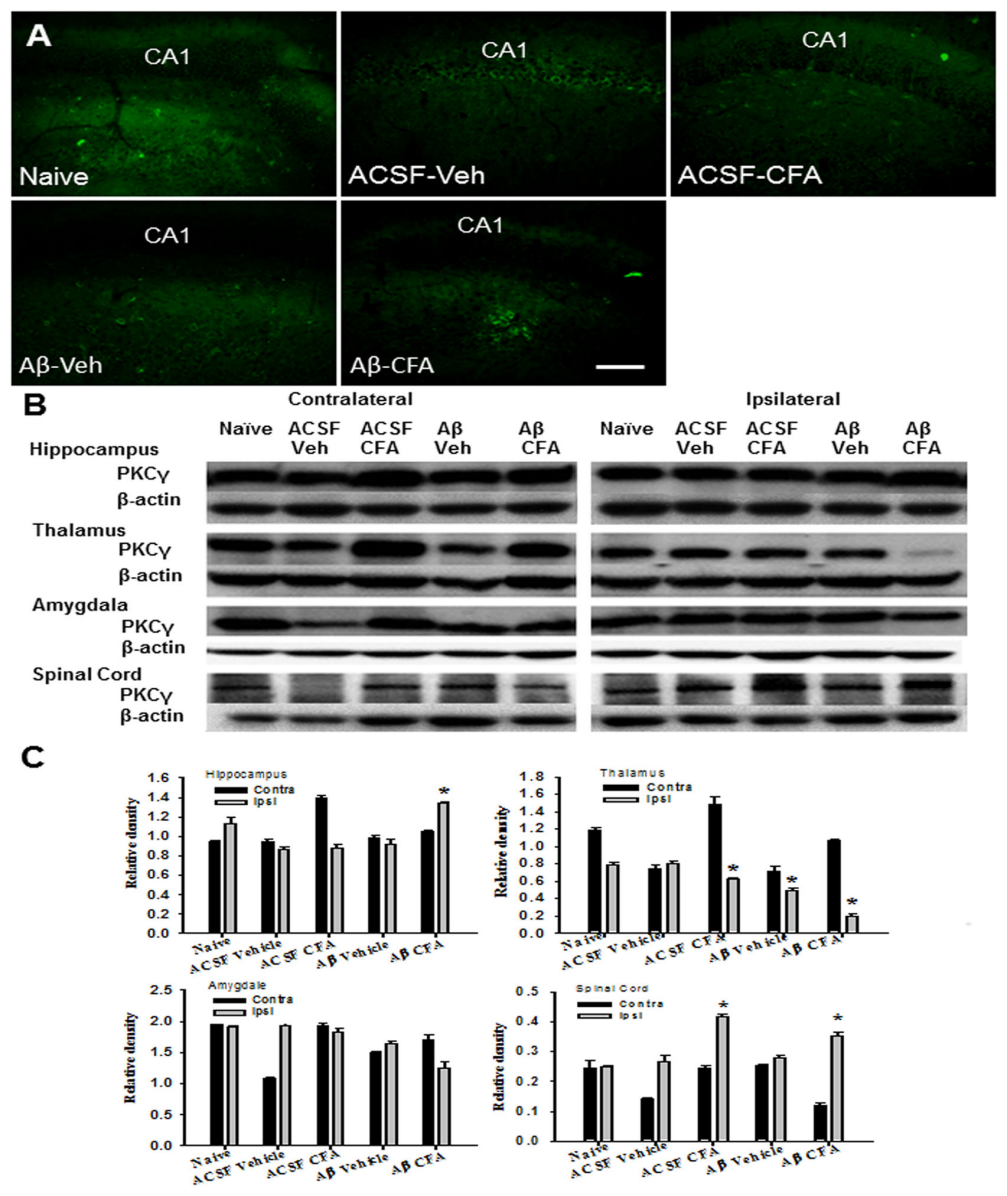
Expression of PKCγ in the brain and spinal cord. **A**–**C**) Immunostaining (A) and Western blot (B, C) showed an upregulation of PKCγ in the hippocampus ipsilateral to ankle inflammation in rats with intra-hippocampal Aβ injection. Scale bar: 100 µm. **B**, **C**) In these same Aβ-injected rats, the PKCγ expression was downregulated in ipsilateral thalamus but not amygdala. In addition, the expression of PKCγ was increased within the spinal cord dorsal horn ipsilateral to ankle inflammation in both intra-hippocampal Aβ- or vehicle-injected rats. * *P*<0.05, as compared with the naïve and ACSF group on the same (ipsilateral) side.

NR1 and PKCγ have been shown to regulate nociceptive behavior [28]. In rats with intra-hippocampal Aβ injection, the expression of NR1 (Figure 5A,B,C) and PKCγ (Figure 6A,B,C) was upregulated in the hippocampus ipsilateral to ankle inflammation as compared to Aβ-injected rats without ankle inflammation (each *P*<0.05). In these same Aβ-injected rats, NR1 (Figure 5B,C) and PKCγ (Figure 6B,C) expression was downregulated on the ipsilateral thalamus, but not amygdala, as compared to Aβ-injected rats without ankle inflammation (each *P*<0.05). In addition, the expression of PKCγ was increased within spinal cord dorsal horn ipsilateral to ankle inflammation in both intra-hippocampal Aβ- or vehicle-injected rats (Figure 6B,C, *P*<0.05). These results indicate that ankle inflammation altered the expression of NR1 and PKCγ within brain regions and that spinal cord dorsal horn implicated in the nociceptive processing and the function of learning and memory.

### Disruption of persistent nociceptive behavior by intra-hippocampal injection of cycloheximide

**Figure 7:**
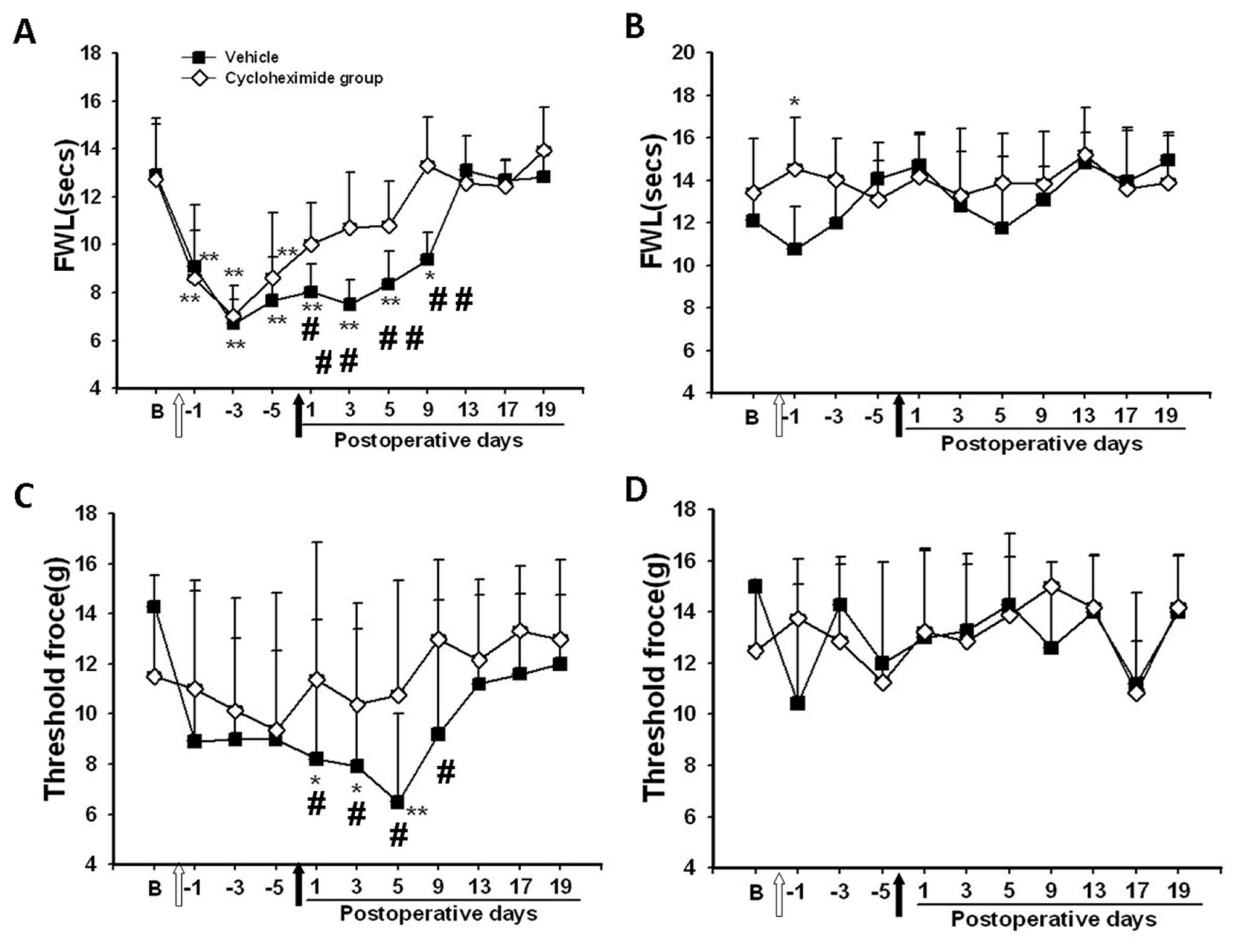
Disruption of persistent nociceptive behavior by cycloheximide. **A**, **C**) Thermal hyperalgesia (A) and mechanical allodynia (C) were developed on the ipsilateral hind paw of the rats with ankle inflammation when tested over a 5-day period (designated as day -1 to -5). The statistical significance (ANOVA, P< 0.05; compared to the baseline) was not marked in the figure for the first five days in order to simplify the presentation. Subsequently, rats treated with cycloheximide showed a swift recovery of both thermal hyperalgesia and mechanical allodynia beginning on day 1 after cycloheximide injection. **B**, **D**) No differences in the nociceptive threshold were detected between cycloheximide and vehicle groups on the contralateral hind paw of these same rats over the entire experimental period. * *P*<0.05, ** *P*<0.01, as compared with baseline threshold of the same group. #*P*<0.05, # # P<0.01, as compared with the cycloheximide group at the same time point. FWL: foot-withdrawal latency. White arrow: injection of CFA into an ankle; Black arrows: injection of saline (1% DMSO in saline) or cycloheximide into the hippocampus.

Thermal hyperalgesia and mechanical allodynia were developed on the ipsilateral hind paw of CFA-injected rats when tested over a 5-day period (designated as day -1 to -5) after CFA injection, as compared with their own baseline (Figure 7A,C, *P*<0.05). To examine whether impairment of learning and memory would influence the recovery of established thermal hyperalgesia and mechanical allodynia, cycloheximide or vehicle was injected into the hippocampal CA1 area and thermal hyperalgesia and mechanical allodynia were again examined on post-injection day 1, 3, 5, 9, 13, 17, and 19. Rats treated with cycloheximide showed a quick recovery of both thermal hyperalgesia and mechanical allodynia beginning on day 1 after the cycloheximide injection, as compared to each corresponding baseline (Figure 7A,C, *P*<0.05). In contrast, the recovery of thermal hyperalgesia and mechanical allodynia did not occur in the vehicle group until post-injection day 9, as compared to each corresponding baseline (Figure 7A,C, *P*<0.05). Neither cycloheximide nor vehicle injection into the hippocampus impaired locomotor activity.

**Figure 8:**
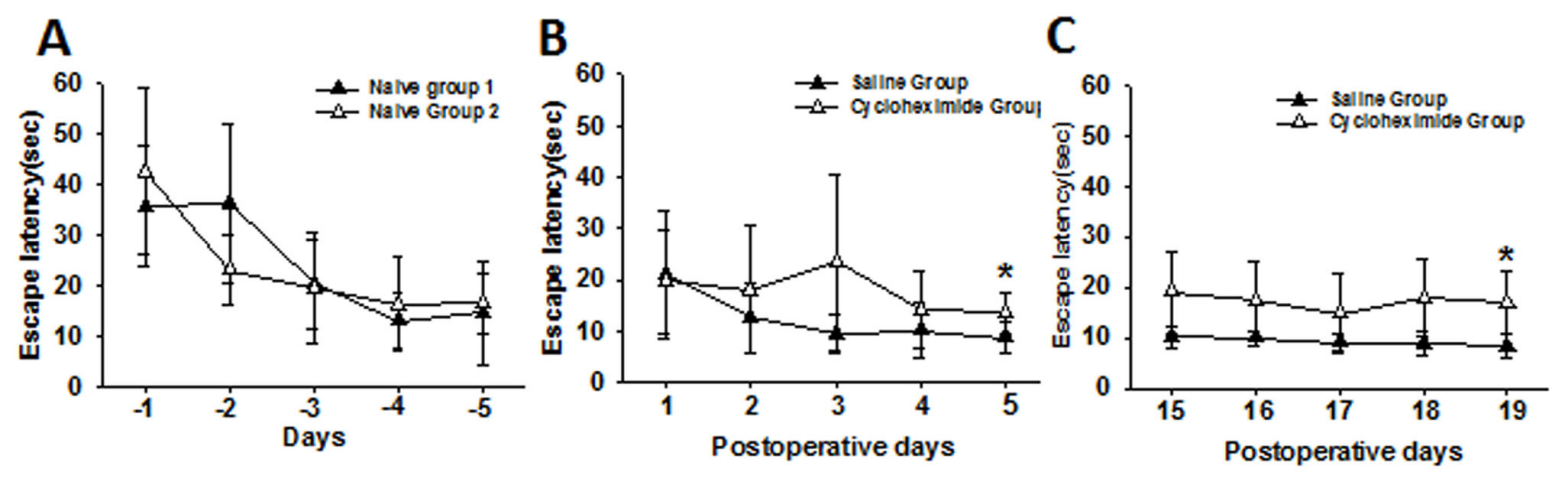
Learning impairment in naïve rats after intra-hippocampal cycloheximide. Intra-hippocampal cycloheximide injection (not saline) disrupted the established spatial learning (**A**: before the injection) in a parallel group of rats without ankle inflammation, as shown in two separate series of the Morris water maze test (**B**: post-injection day 1-5 and **C**: post-injection day 15-19). *P< 0.05, as compared with the intra-hippocampal vehicle group.

The effect of intra-hippocampal cycloheximide injection on the recovery of thermal hyperalgesia and mechanical allodynia was not due to an altered baseline thermal or mechanical nociceptive threshold, because no differences in nociceptive threshold were detected between cycloheximide and vehicle groups on the contralateral hind paw of these same rats over the entire experimental period (Figure 7B,D, *P*>0.05). However, the same intra-hippocampal cycloheximide injection disrupted the established spatial learning in a parallel group of rats without ankle inflammation as demonstrated in two separate series of the Morris water maze test (post-injection day 1-5 and post-injection day 15-19), as compared with intra-hippocampal vehicle group (Figure 8 *P*<0.05). Therefore, this early recovery of thermal hyperalgesia and mechanical allodynia in rats treated with cycloheximide is likely related to the impaired cognitive response similar to that seen in Experiment 1 following the intra-hippocampal Aβ injection.

## Discussion

We conducted two experiments to examine a relationship between learning/memory and nociceptive behaviors. The first experiment examined whether pre-existing learning impairment induced by A-beta would change the course of nociceptive behavior. The second experiment investigated whether the established nociceptive response to ankle inflammation would be reduced by intra-hippocampal injection of cycloheximide (a protein synthesis inhibitor) that disrupted the related nociceptive (pain) memory. Clinical studies indicate that pain response is attenuated in patients with cognitive and learning dysfunction such as AD [7,29–31], although we did not assess performance of short versus long term memories in this study. Nonetheless, our results support the notion that learning impairment could alter the response to persistent nociception, suggesting an important role of cognitive adaptation in the mechanisms of chronic pain.

A number of preclinical models have been used to induce learning impairment including Aβ1-40 and Aβ1-43 [32], Aβ1-40 alone [33], or Aβ1-42 alone [34] cerebral administration. In the first experiment of this study, a mixture of Aβ1-40 and Aβ1-42 was used because it has been shown that a) initial deposition of Aβ1-42 is an early pathological process of senile plaque in the brain of AD, b) mature amyloid plaques were developed following the further deposition of Aβ1-40, and c) the amount of aggregated peptide correlates with the relative ratio of Aβ1-40 versus Aβ1-42 [32]. Consistently, lasting deposition of Aβ was detected in the hippocampus in rats that received the Aβ 1-40/1-42 mixture. Of note is that, while exogenous Aβ was injected into the hippocampus just to establish this model, its expression would not indicate the endogenous Aβ level. Moreover, these same rats showed a lower expression of ChAT in the hippocampus, thalamus, and amygdala as compared with vehicle-treated rats. Since ChAT is an enzyme critical for acetylcholine synthesis, its downregulation suggests a loss of cholinergic neurons and a decrease in acetylcholine release indicative of an early pathological process in AD [27]. Therefore, learning impairment induced by intra-hippocampal Aβ injection mimics that demonstrated in preclinical models of AD [8,9].

In the second experiment, intra-hippocampal injection of cycloheximide was used to examine whether disruption of learning and memory would influence the recovery of nociceptive behavior. Cycloheximide has been shown to inhibit protein synthesis in brain homogenates [35–37] and disrupt learning and memory when administered to chicks in conjunction with a reminder treatment [38]. The effect of cycloheximide on learning and memory consolidation is likely due to its role in blocking translation of polypeptide chains at the elongation and termination phases and provoking the accumulation of ribosome subunits on mRNA leading to inhibition of the protein synthesis [39]. In this experiment, disruption of learning and memory was independently confirmed by the Morris water maze task in rats receiving cycloheximide, but not vehicle, injection into bilateral hippocampal CA1 area, which lasted for at least 19 days after the cycloheximide treatment. Concurrently, the recovery of nociceptive behavior was substantially shortened in those rats treated with cycloheximide, suggesting that the function of learning/memory is contributory to sustaining nociceptive behavior in rats. It should be pointed out that chronic pain may contribute to deficits in working memory without alterations in long-term memory. Both models used in this study would affect memory beyond the working memory phase. Moreover, we did not specifically test performance of short versus long term memories. These issues merit further investigation in future studies.

A number of studies have shown that a state of dysfunctional hippocampus such as that seen in AD due to Aβ deposition could result in failure to recall the location of a hidden-platform in a water maze [40–42]. Similar to the role of the hippocampus in learning and memory, the amygdala participates in the translation of cognitive input into neuroendocrine activity as well [43]. Accordingly, lesions of the amygdala and hippocampus produced emotional disturbances and learning impairment [44,45]. On the other hand, thalamus is critical to the central processing of nociceptive signals [46,47]. Consistent with an important functional role of hippocampus and amygdala in cognition and learning, our results indicate that the expression of NR1 and PKCγ was altered in these brain regions in rats with Aβ-induced learning impairment.

It is well known that an upregulated expression of the NMDA receptor and PKCγ is associated with persistent nociception due to the initiation of intracellular cascades through calcium influx [28,48,49]. Importantly, several studies have shown a relationship between Aβ and NMDA receptor activity as well. For example, 1) Aβ increases excitotoxicity mediated by glutamate receptors including the NMDA receptor [50,51]; 2) a low dose of Aβ can exacerbate a delayed cognitive impairment caused by activation of the NMDA receptor [52]; and 3) Aβ-induced excitotoxicity may be associated with the reduced glutamate uptake [53] and/or increased glutamate release [54,55]. Therefore, at least one of the mechanisms responsible for the attenuation of thermal hyperalgesia and mechanical allodynia in Aβ-injected rats might be related to a modulatory role of Aβ on the NMDA receptor and PKCγ activity within critical brain regions implicated in learning and perception of nociceptive input. Future studies would elucidate the cellular mechanisms of such interactions.

The present data suggest that a learning process may promote cognitive maladaptation due to a state of persistent nociception, which in turn could alter the perception of nociceptive input. When such a learning process was prevented and disrupted by the intra-hippocampal Aβ or cycloheximide administration, the recovery of nociceptive behavior was substantially shortened despite a state of persistent nociception. While in this study disruption of the hippocampal function by injecting either Aβ or cycloheximide was shown to impact both memory and nociceptive behaviors, it remains to be seen whether there is a reciprocal relationship between transient memory impairment and pain experiences. Nonetheless, these results suggest that pharmacological and/or psychological interventions aimed at disrupting cognitive maladaptation to a state of persistent nociception may be a useful approach to preventing and treating chronic pain. Our results also suggest that a preclinical model of combined learning impairment and persistent nociception may be useful to explore the brain mechanisms of the transition from acute to chronic pain.
